# Capturing multidimensionality in stroke aphasia: mapping principal behavioural components to neural structures

**DOI:** 10.1093/brain/awu286

**Published:** 2014-10-20

**Authors:** Rebecca A. Butler, Matthew A. Lambon Ralph, Anna M. Woollams

**Affiliations:** Neuroscience and Aphasia Research Unit, School of Psychological Sciences, Zochonis Building, University of Manchester, Brunswick Street, Manchester, M13 9PL, UK

**Keywords:** lesion symptom mapping, aphasia, language processing, phonology, semantics

## Abstract

Stroke aphasia is a multidimensional disorder in which patient profiles reflect variation along multiple behavioural continua. We present a novel approach to separating the principal aspects of chronic aphasic performance and isolating their neural bases. Principal components analysis was used to extract core factors underlying performance of 31 participants with chronic stroke aphasia on a large, detailed battery of behavioural assessments. The rotated principle components analysis revealed three key factors, which we labelled as phonology, semantic and executive/cognition on the basis of the common elements in the tests that loaded most strongly on each component. The phonology factor explained the most variance, followed by the semantic factor and then the executive-cognition factor. The use of principle components analysis rendered participants’ scores on these three factors orthogonal and therefore ideal for use as simultaneous continuous predictors in a voxel-based correlational methodology analysis of high resolution structural scans. Phonological processing ability was uniquely related to left posterior perisylvian regions including Heschl’s gyrus, posterior middle and superior temporal gyri and superior temporal sulcus, as well as the white matter underlying the posterior superior temporal gyrus. The semantic factor was uniquely related to left anterior middle temporal gyrus and the underlying temporal stem. The executive-cognition factor was not correlated selectively with the structural integrity of any particular region, as might be expected in light of the widely-distributed and multi-functional nature of the regions that support executive functions. The identified phonological and semantic areas align well with those highlighted by other methodologies such as functional neuroimaging and neurostimulation. The use of principle components analysis allowed us to characterize the neural bases of participants’ behavioural performance more robustly and selectively than the use of raw assessment scores or diagnostic classifications because principle components analysis extracts statistically unique, orthogonal behavioural components of interest. As such, in addition to improving our understanding of lesion–symptom mapping in stroke aphasia, the same approach could be used to clarify brain–behaviour relationships in other neurological disorders.

## Abstract (precis)

Butler *et al.* relate behavioural deficits in 31 patients with chronic stroke aphasia to underlying neural structures. Using principal components analysis, they reduce a neuropsychological battery to three independent dimensions: phonological, semantic and executive-cognition. Phonological and semantic processing are linked to dorsal and ventral pathway integrity, respectively

## Introduction

Aphasia is a common consequence of middle cerebral artery stroke. Patterns of preserved and impaired language abilities are highly variable, meaning that post-stroke aphasic individuals form a heterogeneous clinical group. In attempting to relate chronic stroke aphasic performance to underlying neural damage, three challenges must be met: (i) mapping neuropsychological test performance to underlying primary cognitive-language systems (*cf*. Patterson and Lambon Ralph, 1999; Lambon Ralph *et al.*, 2002; Schwartz *et al.*, 2006); (ii) deconstruction of co-occurring deficits within individual participants; and (iii) identification of the neural regions that uniquely support a given ability (made more challenging given that stroke lesions do not sample the brain randomly but are constrained by vascular anatomy (*cf*. Phan *et al.*, 2005). The current study overcame these challenges by adopting a novel approach. Specifically, we applied statistical data reduction techniques to detailed neuropsychological assessments thereby revealing three principal, independent language-cognitive components that could then be related directly to the underpinning neural regions. This technique allowed us to deconstruct the multidimensional nature of chronic stroke aphasia and identify its neural bases more accurately than analyses based upon categorical classifications or individual tests.

Previous behavioural research has identified dissociable semantic and phonological aspects of aphasic performance. Lambon Ralph *et al.* (2002) found that a large proportion of variance in naming accuracy and error types could be accounted for by the integrity of phonological and semantic processing in a case series of 21 aphasic individuals. Likewise, Schwartz *et al.* (2006) demonstrated that a computational model in which lesions were either applied to phonological or semantic components could account for a large proportion of variance in the behavioural performance of 94 aphasic participants. In addition to phonological and semantic factors, general executive-cognitive ability has been found to affect aphasic performance (Jefferies and Lambon Ralph, 2006; Sharp *et al.*, 2010; Robson *et al.*, 2012; Brownsett *et al.*, 2014), as well as influence response to therapy (Lambon Ralph *et al.*, 2010).

At a neuroanatomical level, the distinction between phonological and semantic aspects of aphasic performance could reflect (i) differences in extent of damage to dorsal versus ventral white matter language pathways (Hickok and Poeppel, 2004, 2007; Saur *et al.*, 2008; Ueno *et al.*, 2011; Weiller *et al.*, 2011; Kümmerer *et al.*, 2013); and/or (ii) integrity of perisylvian versus extrasylvian brain regions (Price *et al.*, 1997; Ardila, 1999; Henry *et al.*, 2007). In keeping with these proposals, deficits in conversational speech production have been found to correlate with integrity to perisylvian grey and white matter (Borovsky *et al.*, 2007; Schwartz *et al.*, 2012) whereas phonological repetition is associated with inferior parietal and dorsal language pathways (Fridriksson *et al.*, 2010; Hartwigsen *et al.*, 2013). Likewise, semantic errors in naming performance have been associated with lesions to the anterior middle and superior temporal gyri (Schwartz *et al.*, 2009), occur after electro-stimulation of ventral white matter pathways (Duffau *et al.*, 2005), and emerge as a result of damage to anterior temporal regions responsible for the multimodal, selective semantic impairment seen in semantic dementia (Bozeat *et al.*, 2000; Lambon Ralph *et al.*, 2001; Woollams *et al.*, 2008; Mion *et al.*, 2010).

Here we present a novel approach to isolating different cognitive abilities underlying chronic aphasic performance and to identifying their neural substrates. Based on previous analyses of large-scale case-series data from Alzheimer’s disease and other groups (Lambon Ralph *et al.*, 2002, 2003; Henry *et al.*, 2012; Robson *et al.*, 2012; Kümmerer *et al.*, 2013), detailed behavioural results from a case-series of individuals with heterogeneous chronic stroke aphasic profiles were entered into a principal component analysis (PCA). This data reduction technique extracts the underlying cognitive-language factors which best explain the variation in the data. These statistically-independent (orthogonal) factors were then used in a voxel-wise analysis of the patients’ structural neuroimaging data. This yielded a set of statistical parametric maps showing brain regions where tissue integrity relates to the level of core language-cognitive impairments.

Using PCA factor scores as predictors of lesion data offers a number of important advantages over analyses based upon categorical classifications or individual tests. First, the PCA approach capitalizes on the additional statistical reliability offered by combining data from multiple tests (Lambon Ralph *et al.*, 2002). To take the example of picture naming, a widely used neuropsychological assessment, additional sensitivity and reliability can be obtained by using multiple tests that vary in difficulty (e.g. the Graded Naming Test versus Boston Naming Test versus the 64-item Cambridge Naming Test). The use of harder naming tests ensures sensitivity to mild deficits, whereas the use of easier naming tests avoids floor effects in severe patients. These naming measures are not suitable for simultaneous entry as predictors in a neuroimaging analysis, however, as they are strongly intercorrelated. Secondly, rotated PCA allows deconstruction of each assessment into its cognitive components. For example, as noted in previous studies (Lambon Ralph *et al.*, 2002; Schwartz *et al.*, 2006), picture naming draws on both phonological and semantic processing, which can be extracted and separated by rotated PCA and then each component related to the key underlying patterns of neural damage.

This approach contrasts with more common methods that attempt to relate damaged brain regions to language performance. One approach is to focus on the presence of particular symptoms by considering a particular deficit (e.g. poor picture naming), either by comparing a group with a deficit to healthy controls or by correlating naming scores in a heterogeneous group of patients. Although the latter approach has the advantage of preserving the continuous nature of behavioural performance, both techniques are inherently unidimensional and do not consider the presence of co-occurring deficits (e.g. poor comprehension). Although co-occurring deficits can be covaried out when using continuous scores, if these are strongly correlated with the measure of interest, then the problems of collinearity mean that it is very difficult to isolate areas associated with different component abilities, as any areas associated with multiple abilities will not appear as significant. This can be avoided when using continuous predictors that are uncorrelated, as is the case for rotated PCA factor scores.

Another categorical group-based approach is to relate damage to diagnostic classifications of aphasia type. Classifications from the Boston Diagnostic Aphasia Examination (BDAE) (Goodglass and Kaplan, 1983) or the Western Aphasia Battery (Kertesz, 1982) are useful clinical tools for appraising an individual’s performance profile across a number of assessments (*cf*. the probabilistic approach of the Aachen Aphasia Test, considered further in the ‘Discussion’ section). Although these approaches summarize performance over multiple dimensions, their exclusively categorical nature limits their capacity to identify crucial specific brain regions (see ‘Results’ section; Fig. 3) because the classifications represent a subregion of the multidimensional aphasic space rather than an extraction of the principal dimensions of the space itself. A potentially useful analogy is that of the relationship between colours and the 3D (red-green-blue: RGB) hue space. Colour names, like aphasia classifications, are a handy shorthand for subregions in the RGB space (e.g. pink, violet, orange) but a true understanding of hue requires the colours to be broken down into their constituent parts and their positions along the three principal RGB dimensions to be quantified.

Extending this approach, our key hypothesis is that separable brain regions underpin principal cognitive-language dimensions rather than processing of individual tasks (Patterson and Lambon Ralph, 1999). Accordingly, to test this approach, participants were not recruited on the basis of having specific aphasia types or symptoms. Instead, we recruited a ‘full-range’ clinical sample of individuals with chronic stroke aphasia, with all of the associated behavioural heterogeneity and variation in severity that this approach entails. The mapping of any multidimensional space is more effective if the full space is sampled rather than extracting exemplars from a limited subregion. Like any form of correlation, voxel-wise analysis methods require variance in both the patients’ behavioural measures and regional brain-tissue integrity, which was achieved by avoiding a classification-based sampling method. Statistical sensitivity to regions that support a specific ability requires simultaneous consideration of orthogonal predictors, and this was achieved using rotated PCA factor scores.

## Materials and methods

### Participants

Aphasic participants were recruited from the North West of England via speech and language therapy services and stroke community groups. Participants were included if they had chronic stroke aphasia, i.e. they had an enduring impairment in producing and/or understanding spoken language and were at least 12 months post-stroke at time of scanning and assessment (*n* = 31). All participants were recruited on the basis that they reported one left hemisphere stroke, either ischaemic or haemorrhagic. To align with our sampling requirement, no restrictions were placed according to aphasia type or severity (we recruited from global to minimal aphasia). Participants were excluded if they had any contraindications for scanning, were pre-morbidly left-handed, had more than one stroke, or had any other significant neurological conditions. All participants had English as their first language. For demographic details of participants, see Table 1. Informed consent was obtained from all participants prior to participation under approval from the North West Multi-Centre Research Ethics Committee, UK.

**Table 1:** Participant background information

Patient No.	Initials (BDAE code)	Age (years)	Gender	Years of education	Time post-stroke (months)	BDAE classification
1	DBb (W2)	66	M	12	59	Wernicke
2	ES (G3)	69	M	11	39	Global
3	ESb (G1)	68	M	11	142	Global
4	KW (B5)	81	M	10	24	Broca
5	BS (B4)	59	M	11	103	Broca
6	KL (NF5)	55	M	13	31	Mixed non-fluent
7	LM (G2)	63	M	11	13	Global
8	DB (W1)	60	M	12	44	Wernicke
9	PE (W/C)	73	F	16	22	Wernicke/conduction
10	KS (TSA)	59	M	12	12	TSA
11	KK (B6)	48	M	12	33	Broca
12	WM (NF1)	77	M	11	66	Mixed non-fluent
13	GL (B2)	47	M	12	18	Broca
14	DCS (B7)	45	F	12	12	Broca
15	JSa (NF4)	73	M	11	190	Mixed non-fluent
16	JSc (B8)	78	M	12	76	Broca
17	JA (NF6)	65	M	11	128	Mixed non-fluent
18	JJ (A1)	84	M	12	25	Anomia
19	JM (A7)	62	M	11	110	Anomia
20	JSb (A4)	72	M	11	23	Anomia
21	ER (NF2)	64	F	14	181	Mixed non-fluent
22	HN (A3)	81	M	10	56	Anomia
23	BH (NF3)	64	M	11	26	Mixed non-fluent
24	EB (A8)	61	M	17	12	Anomia
25	DM (B3)	49	M	17	42	Broca
26	DS (TMA)	72	M	11	106	TMA
27	AG (B1)	55	M	11	131	Broca
28	LH (A9)	65	M	11	81	Anomia
29	JMf (A5)	70	F	11	84	Anomia
30	AL (A6)	49	F	12	69	Anomia
31	TJ (A2)	60	M	12	23	Anomia

Cases are ordered according to BDAE severity. TSA = transcortical sensory aphasia; TMA = transcortical motor aphasia.

The healthy control group, which was used in the various neuroimaging analyses, consisted of 19 right-handed healthy older adults (eight females, 11 males), group matched to the patients for age and education: mean age = 68.21 years [standard deviation (SD) = 5.99], range = 59–80 years; mean years of education = 13.06 years (SD = 2.77), range = 10–18 years. For those neuropsychological tasks without published normative data, we collected control data from an age and education group matched subset of the healthy control participants (three females, 10 males): mean age = 68.69 years (SD = 6.55), range = 59–80 years; mean years of education = 12.55 (SD = 2.38), range = 10–17 years.

### Neuropsychology

#### Assessments

In addition to the BDAE (Goodglass and Kaplan, 1983; Goodglass *et al.*, 2000), a battery of language tests was administered to assess the participants’ language and cognitive abilities in a comprehensive fashion. The assessments involved input and output phonological processing, semantic processing and sentence comprehension, as well as more general cognitive function. Assessments were conducted with participants over several testing sessions, with the pace and number per session determined by the participant.

The language assessments included a variety of subtests from the Psycholinguistic Assessments of Language Processing in Aphasia (PALPA) battery (Kay *et al.*, 1992), including: same-different auditory discrimination using non-word minimal pairs (PALPA 1); same-different auditory discrimination using word minimal pairs (PALPA 2); immediate repetition of non-words (PALPA 8); delayed repetition of non-words (PALPA 8); immediate repetition of words (PALPA 9); and delayed repetition of words (PALPA 9). A number of tests from the 64-item Cambridge Semantic Battery (Bozeat *et al.*, 2000) were also included: the spoken word-to-picture matching task; a written word-to-picture matching version of the same task; the picture version of the Camel and Cactus Test; and the picture naming test. To increase sensitivity to mild naming deficits, the 60-item Boston Naming Test (BNT) (Kaplan *et al.*, 1983) was also used. Similarly, to increase sensitivity to subtle semantic deficits, a 96-trial synonym judgement test with words presented in spoken and written form (Jefferies *et al.*, 2009) was also used. To capture syntax level deficits, the spoken sentence comprehension task from the Comprehensive Aphasia Test (CAT) (Swinburn *et al.*, 2005) was administered. Although we included this and subtests from the BDAE (including the Cookie Theft description) as assessments of discourse level processing, the focus of our analysis in this study was on deficits at the single word processing level, as these are the building blocks of language and involve tasks that are sensitive to residual abilities even in severe cases (Henseler *et al.*, 2014). The additional cognitive tests included forward and backward digit span (Wechsler, 1987), the Brixton Spatial Rule Anticipation Task (Burgess and Shallice, 1997), and Raven’s Coloured Progressive Matrices (Raven, 1962).

On language assessments, apart from the Comprehensive Aphasia Test sentence comprehension test (Swinburn *et al.*, 2005), participants were scored on their first response. For the Comprehensive Aphasia Test test, two points are given for a correct response and one point is given for delayed correct responses or self-corrections. For the two naming assessments, participants’ responses were marked correct if they were given within 5 s of presentation. Minor articulatory dysfluencies, but not phonological errors, in responses were accepted as correct. Repetition of auditory stimuli was provided if requested by participants.

#### Principal components analysis

Participants’ scores on all assessments were entered into a PCA with varimax rotation (conducted with SPSS 16.0). There is no clear guide on the number of cases needed for PCA, but good results have been obtained with a subject to variable ratio of 1.2 (Barrett and Kline, 1981). We had 17 variables and 31 cases, making the ratio in the current study 1.8. In addition, Preacher and MacCallum (2002) suggest factor recovery is good beyond a sample size of 20. Our same size therefore seems adequate for the purposes of PCA.

Factors with an eigenvalue ≥1.0 were extracted and then rotated. After orthogonal rotation, the factor loadings of each test allowed interpretation of what cognitive-language primary process was represented by that factor. Individual participants’ scores on each extracted factor were then used as behavioural covariates in the neuroimaging analysis.

### Neuroimaging

#### Acquisition and processing

High resolution structural T_1_-weighted MRI scans were acquired on a 3.0 T Philips Achieva scanner (Philips Healthcare) using an 8-element SENSE head coil. A T_1_-weighted inversion recovery sequence with 3D acquisition was used, with the following parameters: repetition time = 9.0 ms, echo time = 3.93 ms, flip angle = 8°, 150 contiguous slices, slice thickness = 1 mm, acquired voxel size 1.0 × 1.0 × 1.0 mm^3^, matrix size 256 × 256, field of view = 256 mm, inversion time = 1150 ms, SENSE acceleration factor 2.5, total scan acquisition time = 575 s.

Participants’ MRI scans were normalized and segmented using a modified unified segmentation-normalization procedure optimized for lesioned brains (Seghier *et al.*, 2008) implemented in Statistical Parametric Mapping (SPM) 8 (Wellcome Trust Centre for Neuroimaging, http://www.fil.ion.ucl.ac.uk/spm/) running under Matlab 2009a. Images were then smoothed with an 8 mm full-width at half-maximum Gaussian kernel and used in the lesion analyses described below. As suggested by Geva *et al.* (2012), the analyses were conducted on the normalized images incorporating both grey and white matter to allow detection of both cortical and subcortical correlates of deficits.

#### Automated lesion identification procedure

Automated outlines of brain areas classified as ‘abnormal’ were generated using Seghier *et al.*’s (2008) modified segmentation-normalization procedure. Data from all participants with stroke aphasia and all healthy controls were entered into the segmentation-normalization. Segmented images were smoothed with an 8 mm full-width at half-maximum Gaussian kernel as recommended by Seghier *et al.* (2008) and submitted to the automated routine’s lesion identification and definition modules using the default parameters apart from the lesion definition ‘U-threshold’, which was set to 0.5. We modified the U-threshold from 0.3 to 0.5 after comparing the results obtained for a sample of patients to what would be nominated as lesioned tissue by an expert neurologist. The generated images were used to create the ‘lesion’ overlap map in Fig. 2 and the individual ‘lesion’ outlines in Fig. 7.

Although it has been demonstrated that cost-function masking with a hand-traced lesion mask is the optimal method for spatial normalization of lesioned brains (Andersen *et al.*, 2010; Wilke *et al.*, 2011), this technique is both labour intensive and somewhat subjective as to what abnormalities fall within the lesion boundaries. We were interested in adopting an efficient and objective method of ‘lesion’ identification for use with large sample of patients, therefore we selected the fully automated method developed by Seghier *et al.* (2008). This method has been shown to perform at an acceptable level relative to hand tracing (Wilke *et al.*, 2011), particularly in the case of large lesions, as was true of the majority of patients in our sample. The automated method involves initial segmentation and normalization into tissue classes of grey and white matter, CSF and an ‘extra’ tissue class, which allows for the presence of the ‘lesion’. After smoothing, voxels that emerge as outliers relative to normal participants are identified and the union of these outliers provides the ‘fuzzy lesion map’, from which is derived the lesion outline. It should be emphasized that this method essentially identifies areas of neural abnormality rather than ‘lesion’ *per se*. It is therefore likely to be affected by the abnormal shape of the ventricles in patients with large lesions, and hence is used with the caveat that periventricular results are treated with caution (Geva *et al.*, 2012). On the other hand, this procedure has the potential to be sensitive to indirect lesion effects that would be missed using hand tracing (Wilke *et al.*, 2011). Ultimately, our decision to adopt an automated lesion identification procedure in this study was driven by a desire to use a method that was easily replicable and that would effectively scale up to larger patient samples.

### Voxel-based morphometry

In assessing brain–behaviour relationships, a number of options exist. One widely used technique is voxel-based lesion–symptom mapping (VLSM) (Bates *et al.*, 2003). VLSM binarizes each patient’s lesion map and then compares behavioural scores of those patients with a ‘damaged’ voxel against individuals with an ‘intact’ voxel. This widely used procedure has the asset of preserving the continuous nature of behavioural scores, but it also dichotomizes brain integrity and is limited to coverage of lesioned areas. We chose to use another widely-used technique for lesion symptom mapping, voxel-based morphometry (VBM), which indexes neural integrity in the form of continuous voxel intensity values (Ashburner and Friston, 2000). This approach has the advantage of preserving the continuous nature of neural structure and offers whole brain coverage, so is potentially sensitive to areas of Wallerian degeneration remote to the lesion (Geva *et al.*, 2012).

VBM has often been used to compare groups of participants, such as patients with some kind of deficit versus healthy controls (Josephs *et al.*, 2006). This approach therefore dichotomizes behavioural performance and, to illustrate the limitations of this approach in the context of lesion–symptom mapping in chronic aphasia, we provide an example at both the syndrome and symptom levels (see below). VBM has also been used to detect neural correlates of a particular ability by entering test scores as continuous variables (Mummery *et al.*, 2000), a technique also known as voxel-based correlational methodology (VBCM; Tyler *et al.*, 2005). In a direct comparison of VLSM and VBCM in terms of lesion-deficit relationships in a group of 20 chronic stroke aphasic patients, Geva *et al.* (2012) demonstrated that VLSM was more sensitive to non-linear relationships, whereas VBCM was more sensitive to linear relationships. Although the nature of underlying lesion-deficit relationship in the present sample is not known, we opted for VBCM because this approach has the virtue of preserving the continuous nature of both behavioural and neural indices. Graded measures of neural integrity may well be most appropriate when considering other patient populations with different aetiologies (e.g. neurodegenerative conditions).

#### Syndromes and symptoms

The VBM analyses of BDAE subtypes and symptom groups (Figs 3 and 4) were conducted in SPM8 running on Matlab 2009*a* and 2012*a*, respectively. Smoothed and normalized T_1_-weighted images from each patient in the relevant group and from the group of 19 healthy older control participants were entered into the analysis. Statistical comparisons were then carried out between the subtype or symptom group and the control group for every brain voxel. The resulting images show clusters of voxels in which the subtype or symptom group had a significantly lower concentration of tissue than the control group.

The syndrome analysis considered the nine patients with a BDAE classification of anomic aphasia, the eight patients with a classification of Broca’s aphasia and the six patients with a classification of mixed non-fluent aphasia. The symptom analysis considered those patients with the nine lowest scores on the Cambridge picture naming test, the eight lowest scores on delayed non-word repetition, and the six lowest scores spoken word-to-picture matching (numbers in each group were selected to match those falling into various syndromes).

#### Principle component analysis factors and test scores

The VBCM analyses of PCA factors and individual test scores were conducted in SPM8 running on Matlab 2009*a* and 2012*a*, respectively, with sets of factors or scores entered simultaneously as continuous behavioural covariates. The outcome of the analyses therefore denote which voxels’ variation in tissue concentration corresponds to the unique variance in a given principle component or test, while controlling for variation in the other components or tests included in that analysis.

The first analysis used the three continuous multidimensional predictors of the PCA factor scores, which are necessarily uncorrelated (orthogonal) with one another. We then contrasted these results with those obtained on the basis of a non-PCA selection of individual tests that seem to tap the same underlying abilities. Lastly, we contrasted these results with those obtained using individual tests, selected on the basis that they had the highest loadings on each PCA factor.

## Results

### Neuropsychological profiles and principal language-cognitive factors

Participants’ scores on the behavioural assessment battery are given in Table 2, with participants ordered according to their performance on the Boston Naming Test. The heterogeneity of the cohort is evident from participants’ broad range of scores on the assessment battery. These spanned from individuals who performed poorly on all tests in the assessment battery (e.g. Patient DBb) to those who only fell below normal limits on the more demanding, sentence-level assessment (e.g. Patient JMf).

**Table 2:** Participants’ scores on the behavioural assessment battery

ID	Non-word Repetition: Immediate	Non-word Repetition: Delayed	Word Repetition: Immediate	Word Repetition: Delayed	64-Item Naming	Boston Naming Test	Non-word Minimal Pairs	Word Minimal Pairs	Spoken Word to Picture Matching	Written Word to Picture Matching	CAT Spoken Sentence Comprehension	96 Synonym Judgement	Camel and Cactus Test: Pictures	Brixton Spatial Anticipation Test^a^	Raven's Coloured Progressive Matrices^b^	Forward Digit Span^a^	Backward Digit Span^a^	PHON F1	SEM F2	COG F3
**DBb**	**0.00**	**0.00**	**37.50**	**0.00**	**0.00**	**0.00**	**22.22**	**52.78**	**57.81**	**31.25**	**12.50**	**48.96**	**53.13**	**38.18**	**30.56**	**25.00**	**0.00**	−0.33	−2.32	−2.02
**ES**	**0.00**	**0.00**	**0.00**	**0.00**	**4.69**	**0.00**	**48.61**	**54.17**	**78.13**	**90.63**	**25.00**	**72.92**	**73.44**	**40.00**	66.67	**0.00**	**0.00**	−1.67	−0.27	−0.33
**ESb**	**0.00**	**0.00**	**0.00**	**0.00**	**0.00**	**0.00**	**54.17**	**50.00**	**87.50**	**60.94**	**34.38**	**52.08**	**43.75**	**23.64**	38.89	**0.00**	**0.00**	−1.08	−0.70	−1.91
**KW**	**0.00**	**0.00**	**3.75**	**0.00**	**1.56**	**0.00**	**75.00**	**65.28**	**95.31**	**92.19**	**84.38**	**82.29**	89.06	50.91	80.56	**50.00**	42.86	−1.21	−0.20	0.97
**BS**	**3.33**	**0.00**	**5.00**	**1.25**	**3.13**	**1.67**	**65.28**	**75.00**	**92.19**	100.00	**31.25**	**78.13**	**84.38**	**38.18**	91.67	**0.00**	**0.00**	−1.94	0.27	0.68
**KL**	**0.00**	**0.00**	**6.25**	**0.00**	**4.69**	**1.67**	**75.00**	**77.78**	**92.19**	98.44	**28.13**	**68.75**	**78.13**	61.82	88.89	**0.00**	**0.00**	−1.81	−0.07	0.97
**LM**	**13.33**	**3.33**	**27.50**	**0.00**	**1.56**	**1.67**	**43.06**	**54.17**	**67.19**	**53.13**	**28.13**	**57.29**	**68.75**	**32.73**	61.11	**0.00**	**0.00**	−0.87	−1.68	−0.77
**DB**	70.00	**30.00**	**85.00**	**83.75**	**7.81**	**8.33**	87.50	**58.33**	**64.06**	**76.56**	**31.25**	**59.38**	**82.81**	**40.00**	86.11	**37.50**	**14.29**	0.32	−2.30	0.59
**PE**	**13.33**	**3.33**	**45.00**	**41.25**	**20.31**	**11.67**	**77.78**	86.11	96.88	100.00	**50.00**	**79.17**	**84.38**	**41.82**	80.56	**25.00**	28.57	−1.07	0.36	0.46
**KS**	73.33	80.00	93.75	95.00	**31.25**	**13.33**	94.44	95.83	**71.88**	**67.19**	**84.38**	**84.38**	**68.75**	52.73	86.11	100.00	57.14	1.73	−2.40	0.72
**KK**	**33.33**	**3.33**	**56.25**	**26.25**	**42.19**	**15.00**	**72.22**	95.83	**93.75**	**95.31**	**46.88**	**81.25**	**84.38**	76.36	100.00	**0.00**	**0.00**	−1.21	0.16	1.32
**WM**	**36.67**	**30.00**	**55.00**	**41.25**	**39.06**	**25.00**	**47.22**	**63.89**	**92.19**	**75.00**	**50.00**	**61.46**	**51.56**	**43.64**	61.11	**37.50**	28.57	−0.04	−0.53	−1.34
**GL**	93.33	63.33	100.00	**81.25**	**68.75**	**31.67**	98.61	97.22	96.88	**95.31**	**65.63**	**75.00**	**73.44**	58.18	91.67	**37.50**	28.57	0.62	−0.36	0.58
**DCS**	**40.00**	56.67	**72.50**	**68.75**	**67.19**	**43.33**	97.22	97.22	100.00	98.44	**93.75**	91.67	95.31	81.82	100.00	62.50	57.14	0.21	0.05	1.68
**JSa**	**30.00**	**3.33**	**75.00**	**65.00**	**62.50**	**46.67**	**75.00**	**77.78**	**92.19**	98.44	**59.38**	**81.25**	**76.56**	67.27	83.33	**50.00**	28.57	−0.32	0.31	0.23
**JSc**	**36.67**	63.33	**90.00**	91.25	**71.88**	**53.33**	**75.00**	86.11	98.44	98.44	**75.00**	**76.04**	**82.81**	**43.64**	77.78	62.50	42.86	0.42	0.36	−0.26
**JA**	**36.67**	**40.00**	**85.00**	**78.75**	**79.69**	**63.33**	90.28	95.83	100.00	98.44	**78.13**	**63.54**	87.50	61.82	80.56	**37.50**	**0.00**	−0.12	0.59	0.31
**JJ**	**36.67**	**23.33**	**82.50**	**73.75**	**85.94**	**63.33**	**51.39**	**80.56**	98.44	98.44	**56.25**	93.75	**53.13**	**43.64**	41.67	62.50	42.86	0.24	1.43	−2.20
**JM**	83.33	83.33	100.00	98.75	**81.25**	**63.33**	93.06	95.83	100.00	100.00	100.00	**82.29**	**81.25**	76.36	94.44	**50.00**	57.14	0.99	−0.05	0.86
**JSb**	63.33	**36.67**	**86.25**	**81.25**	**75.00**	**63.33**	**76.39**	88.89	**93.75**	**90.63**	**84.38**	**75.00**	**78.13**	60.00	86.11	62.50	28.57	0.47	0.01	0.12
**ER**	**53.33**	**36.67**	**70.00**	**81.25**	**71.88**	**65.00**	81.94	88.89	**95.31**	**93.75**	**56.25**	**84.38**	90.63	**41.82**	38.89	**25.00**	**0.00**	−0.24	1.34	−1.09
**HN**	**36.67**	**23.33**	**83.75**	**80.00**	**65.63**	**65.00**	**77.78**	**76.39**	**93.75**	**93.75**	**37.50**	**85.42**	**85.94**	**25.45**	75.00	**50.00**	42.86	−0.09	0.71	−0.69
**BH**	86.67	80.00	100.00	96.25	95.31	**66.67**	93.06	94.44	98.44	**93.75**	**78.13**	**83.33**	**73.44**	67.27	66.67	62.50	57.14	1.20	0.22	−0.30
**EB**	83.33	**53.33**	100.00	100.00	**81.25**	**66.67**	94.44	98.61	98.44	100.00	**71.88**	94.79	90.63	80.00	100.00	75.00	57.14	0.76	0.17	1.15
**DM**	**60.00**	**10.00**	**73.75**	**68.75**	**75.00**	**71.67**	80.56	93.06	98.44	98.44	**56.25**	95.83	98.44	50.91	91.67	**37.50**	**0.00**	−0.61	1.27	0.42
**DS**	**56.67**	**33.33**	**88.75**	91.25	**84.38**	**73.33**	**79.17**	**77.78**	100.00	100.00	87.50	93.75	89.06	72.73	72.22	**50.00**	28.57	0.13	1.05	0.04
**AG**	73.33	83.33	**77.50**	**87.50**	**87.50**	78.33	100.00	98.61	100.00	100.00	87.50	**89.58**	**75.00**	56.36	75.00	100.00	100.00	1.42	0.21	−0.06
**LH**	**56.67**	**50.00**	**82.50**	88.75	**81.25**	78.33	95.83	97.22	96.88	100.00	90.63	92.71	87.50	76.36	88.89	87.50	57.14	0.70	0.35	0.81
**JMf**	93.33	66.67	96.25	98.75	96.88	80.00	90.28	95.83	100.00	100.00	**71.88**	91.67	93.75	50.91	83.33	62.50	57.14	0.81	0.74	0.11
**AL**	90.00	90.00	100.00	98.75	**93.75**	88.33	91.67	100.00	100.00	100.00	**84.38**	93.75	**79.69**	60.00	91.67	87.50	85.71	1.45	0.26	0.23
**TJ**	93.33	83.33	98.75	92.50	95.31	95.00	87.50	98.61	98.44	100.00	**68.75**	**88.54**	**70.31**	52.73	50.00	75.00	28.57	1.14	1.03	−1.29

Cases are ordered according to BDAE severity. Scores are given as percentages. Scores marked in bold fall below the cut-off for normal performance. The cut-off was calculated as 2 SD below the mean performance (see text for details). ^a^Cut-off based on published norms. ^b^No cut-off available. PHON = phonological factor; SEM = semantic factor; COG = cognitive factor.

#### Identifying principal language-cognitive factors

The rotated PCA produced a three factor solution which accounted for 82% of variance in participants’ performance (F1 = 61%; F2 = 14%, F3 = 7%). The factor loadings of each of the different behavioural assessments are given in Table 3, with individual participants’ scores on each factor provided in Table 2. Tasks which tapped input and/or output phonology (e.g. non-word repetition, minimal pairs, picture naming and also digit span, which involves repetition of strings of numbers) loaded heavily on Factor 1, hence we refer to this factor as ‘Phonology’. Factor 2 was interpreted as ‘Semantics’, as the assessments that loaded heavily on it were those involving processing of meaning, whether receptive or expressive (e.g. spoken word-to-picture matching, synonym judgement and picture naming). Note that the two naming assessments loaded heavily on both of these factors, as they clearly require intact phonological and semantic processing to be performed successfully, consistent with previous results (Lambon Ralph *et al.*, 2002; Schwartz *et al.*, 2006). The assessments that loaded heavily on Factor 3 were more diverse. Both Raven’s progressive matrices and Brixton spatial anticipation taps pattern detection and prediction abilities. Although the Camel and Cactus Test does require semantic knowledge as shown by its disruption in semantic dementia (Bozeat *et al.*, 2000), in this sample, performance seems to be affected more by the ability to reason out the basis for association, which is consistent with the semantic control deficits reported in stroke aphasia (Head, 1926; Jefferies and Lambon Ralph, 2006). We presume the loading for minimal pairs is due to the need for patients with phonological processing deficits to adopt an explicit comparative strategy and problem-solving for this task, which will be most apparent for non-words where semantics provides no support and thus the task becomes very challenging for aphasic patients. Overall, the tests loading on the third factor involve modality-independent choice, discrimination or reasoning, hence it was interpreted as the ‘executive-cognition’ factor.

**Table 3:** Loadings of behavioural assessments on factors extracted from the rotated PCA

	Factor 1 Phonology	Factor 2 Semantics	Factor 3 Cognition
Minimal Pairs – Non-words	**0.581**	0.302	**0.642**
Minimal Pairs – Words	**0.600**	0.498	0.472
Immediate Repetition – Non-words	**0.868**	0.189	0.188
Delayed Repetition – Non-words	**0.917**	0.116	0.142
Immediate Repetition – Words	**0.872**	0.247	0.122
Delayed Repetition – Words	**0.868**	0.336	0.162
64-Item Naming	**0.725**	**0.646**	0.051
Boston Naming Test	**0.688**	**0.649**	−0.094
Spoken Word to Picture Matching	0.206	**0.865**	0.259
Written Word to Picture Matching	0.129	**0.799**	0.488
96 Synonym Judgement	0.414	**0.665**	0.364
Camel and Cactus Test: Pictures	0.011	0.419	**0.731**
CAT Spoken Sentence Comprehension	**0.681**	0.339	0.413
Brixton Spatial Anticipation Test	0.357	0.241	**0.654**
Raven's Coloured Progressive Matrices	0.090	0.057	**0.938**
Forward Digit Span	**0.882**	0.139	0.132
Backward Digit Span	**0.764**	0.132	0.219

Factor loadings >0.5 are given in bold. CAT = Comprehensive Aphasia Test.

#### Capturing global severity

When the behavioural data were entered into an unrotated PCA, all tests in the battery loaded heavily on the first unrotated factor, a factor that can be interpreted as reflecting each participant’s overall aphasic severity. This unrotated ‘severity’ factor correlated highly with the phonological factor from the rotated PCA (*r* = 0.766, *P* < 0.0005), and to a lesser extent with the semantic and cognitive factors (*r* = 0.500, *P* = 0.004 and *r* = 0.405, *P* = 0.024, respectively). This suggests that in this group of individuals with chronic stroke aphasia, severity maps quite closely onto the level of phonological processing impairment.

#### Relationship to aphasia subtypes

**Figure 1:**
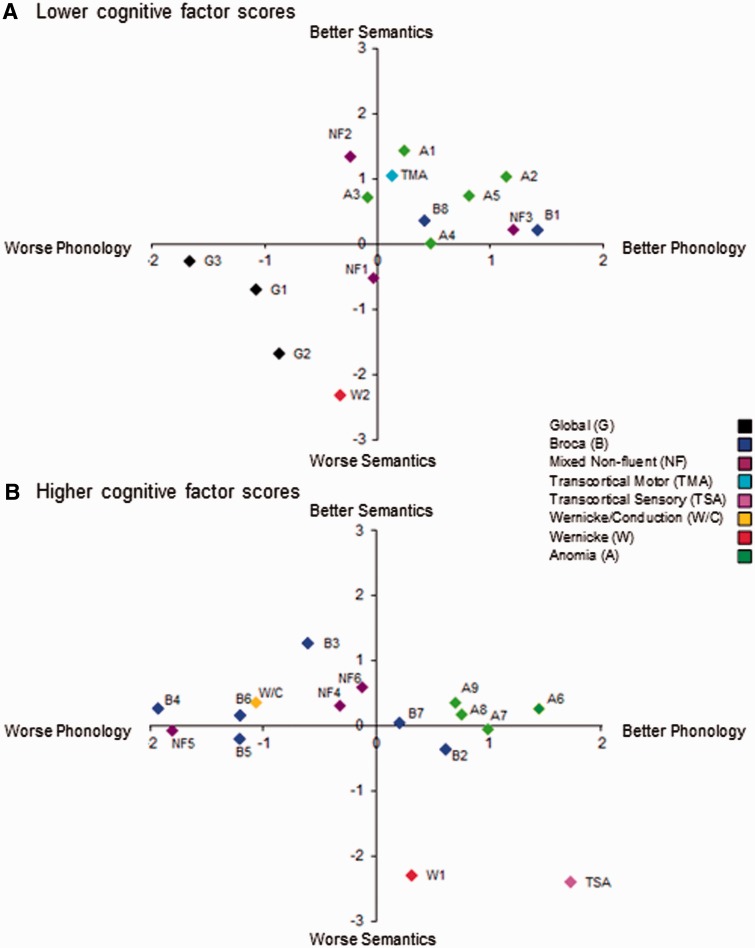
Participants’ scores on phonological and semantic factors, split according to scores on the cognitive factor (above versus below the median). (**A**) Participants with lower scores on the cognitive factor; (**B**) participants with higher scores on the cognitive factor. Dual colour and letter coding reflects each participant’s aphasia classification (Table 1).

Figure 1 shows the relationship between the three factor scores and the BDAE classifications. As noted in the ‘Introduction’ section, each aphasia classification sits within a specific subregion of the 3D PCA space (as colours do within an RGB hue space). Thus, for example, patients with global aphasia are situated in the lower left quadrant of Fig. 1A, indicating poor phonological, semantic and cognitive performance. In contrast, the only participant in the cohort with transcortical sensory aphasia is found in the lower right quadrant of Fig. 1B, reflecting a combination of good phonological and cognitive skills yet impaired semantic performance.

In contrast to a pure categorical organization, it is well known that: (i) within each BDAE subtype, there is considerable variability between individual patients; (ii) there seems to be graded relationships between some subtypes (e.g. Wernicke-to-conduction aphasia); and (iii) many patients do not fit into a specific category (and are given a ‘mixed aphasia’ label). Again, this is like the colour analogy set out above: there are a variety of pinks, oranges and violets; some colours seem to border with each other (e.g. pink-to-red; yellow-to-orange, etc.); and some colours are hard to categorize (e.g. grey or khaki). The three extracted factor scores capture these same graded patterns. For example, the participants with Broca’s aphasia show varying phonological performance yet little variation in their semantic performance. Likewise, the ‘mixed’ non-fluent cases sit in the middle of the three factor space (i.e. with a moderate level of all three impairments). Consequently, by shifting away from a categorical model of aphasia towards a continuous multi-dimensional characterization, these more graded aspects of aphasia are captured while preserving the core differences between prototypical examples of each aphasia type. A key hypothesis for this study was that these continuous and independent factor scores would map more precisely onto key underlying neural regions than alternative categorical and/or unidimensional approaches, which was tested in the next analyses.

### The neural basis of performance in chronic stroke aphasia

#### Lesion overlap

**Figure 2:**
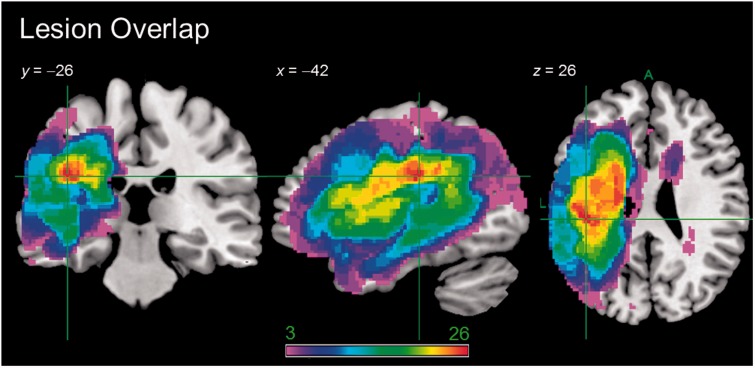
Lesion overlap map showing the distribution of participants’ lesions (*n* = 31). Lesions were identified using Seghier *et al.*’s (2008) automated software. Colour scale indicates number of participants with a lesion in that voxel.

A lesion overlap map for stroke aphasic participants is provided in Fig. 2, and primarily covers the large left hemisphere area supplied by the middle cerebral artery (Phan *et al.*, 2005). All neuroimaging results are shown overlaid on the Ch2better template in MRIcron (Rorden *et al.*, 2007). The maximum number of participants who had a lesion in any one voxel was 26, in the region of the left rolandic operculum.

#### Voxel-based morphometry of syndromes and symptoms

**Figure 3:**
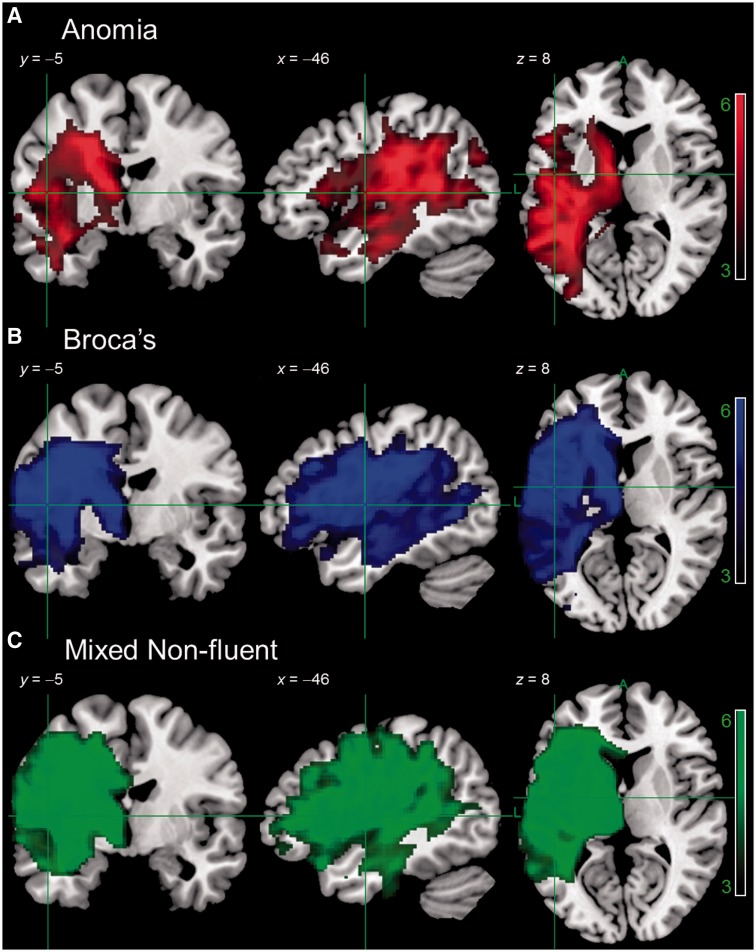
Results of a VBM analysis comparing tissue concentration of participants with anomic aphasia (*n* = 9), cluster size 25 668, Broca’s aphasia (*n* = 8), cluster size 37 447, or mixed non-fluent aphasia (*n* = 6), cluster size 41 357, to healthy older controls (*n* = 19). Image threshold (*t*) 3.0–6.0. Results are presented at *P* < 0.001 voxel-level, *P* < 0.001 FWE-corrected cluster-level. Analyses were not conducted for aphasic subgroups with *n* < 5 participants (global = 3, transcortical motor aphasia = 1, transcortical sensory aphasia = 1, Wernicke’s = 2, Wernicke’s/conduction = 1).

**Figure 4:**
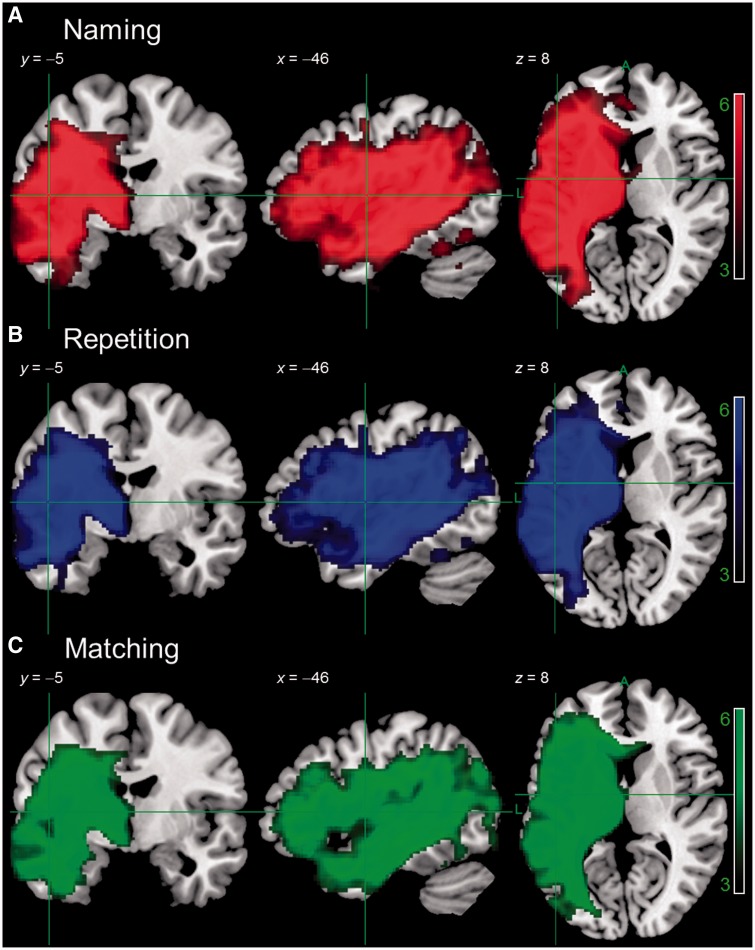
Results of a VBM analysis comparing tissue concentration of participants with the lowest scores on picture naming (*n* = 9), cluster size 51 996, delayed non-word repetition (*n* = 8), cluster size 45 843, or spoken word-to-picture matching (*n* = 6), cluster size 46 834, to healthy older controls (*n* = 19). Image threshold (*t*) 3.0–6.0. Results are presented at *P* < 0.001 voxel-level, *P* < 0.001 FWE-corrected cluster-level. Numbers in each group were chosen for comparability with BDAE Subtypes in Fig. 3.

Results are thresholded at *P* < 0.001 voxel-level, *P* ≤ 0.001 family-wise error (FWE) corrected cluster-level. For the syndrome level analysis, Fig. 3 shows areas of significantly lower tissue concentration in subsets of our participants, as defined by BDAE aphasia classification. Although this approach is multidimensional in the sense that each classification represents a profile of performance over a number of tests, it is nevertheless categorical. Despite some variation across categories, the principal finding was that there was a large lesion overlap between subtypes. This is consistent with previous studies that have used similar approaches (Kümmerer *et al.*, 2013) and presumably reflects the fact that certain brain regions are more likely than others to be affected by a middle cerebral artery stroke (Phan *et al.*, 2005). A symptom level analysis took comparable samples of patients scoring the lowest on individual tests of Cambridge picture naming, delayed nonword repetition and spoken word-to-picture matching (Fig. 4). The results showed even larger overlaps between groups, demonstrating the limitations of a unidimensional categorical approach. Indeed, the VBM analysis for each aphasia subtype and behavioural symptom closely mirrors the lesion overlap map (Fig. 2).

#### Voxel-based morphometry of principle component analysisfactors and test scores

**Figure 5:**
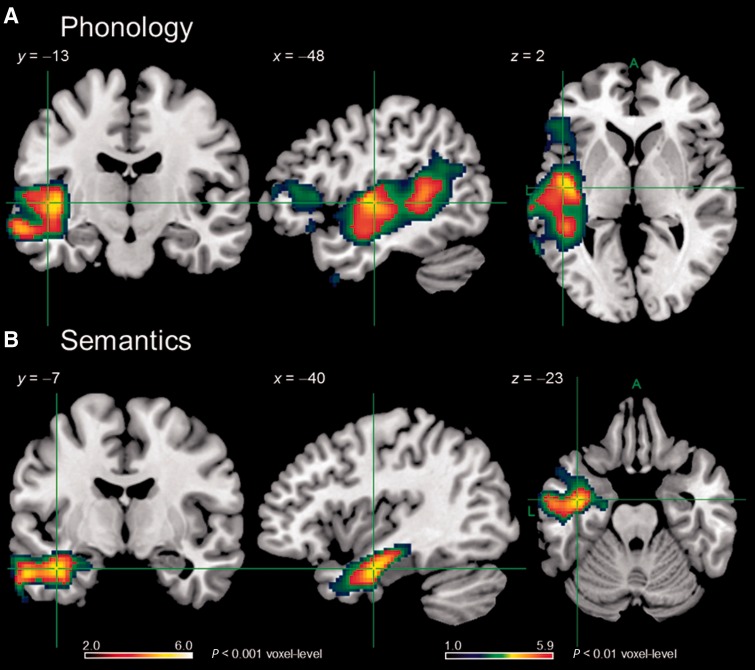
Regions found to relate significantly and uniquely to phonological (**A**) and semantic (**B**) performance in VBCM analyses. Hot overlays are clusters significant at *P* < 0.001 voxel-level, *P* ≤ 0.001 FWE-corrected cluster-level and which were interpreted in the text. Cluster sizes 2622 (**A**) and 856 (**B**) voxels. Image threshold (*t*) 2.0–6.0. ACTC (blue/green) overlays are clusters significant at *P* < 0.01 voxel-level, *P* ≤ 0.001 FWE-corrected. Image threshold (*t*) 1.0–5.9.

For the PCA factor analyses, the VBCM results are shown in Fig. 5. Each map shows where tissue concentration covaries uniquely with a given factor score, which are necessarily uncorrelated with each other. Results are thresholded at *P* < 0.001 voxel-level, *P* ≤ 0.001 FWE corrected cluster-level. Performance on the phonological factor was uniquely correlated with voxels across a number of left hemisphere regions, principally a cluster containing: primary auditory cortex (Brodmann areas 41 and 42); mid to posterior middle and superior temporal gyri; superior temporal sulcus; and posterior portions of the insula, Heschl’s gyrus and the planum temporale. A second cluster in the left inferior prefrontal region was also identified at a slightly lower statistical threshold (Fig. 5A). The phonological cluster also overlapped with white matter regions, the location which encompasses part of the arcuate fasciculus, a key aspect of the dorsal language pathway (Wise, 2003; Catani and ffytche, 2005; Catani *et al.*, 2005; Duffau *et al.*, 2005; Parker *et al.*, 2005; Saur *et al.*, 2008).

Performance on the semantic factor was uniquely related to a cluster of voxels in the left hemisphere anterior temporal lobe (Fig. 5B). The cluster overlapped with the anterior middle temporal gyrus and the temporal stem (including the dorsal edges of the inferior temporal gyrus and fusiform gyrus). Thus, with regards to white matter, the cluster included an area corresponding to part of the ventral language route, overlapping parts of the inferior longitudinal fasciculus, inferior fronto-occipital fasciculus and uncinate fasciculus (e.g. Wise, 2003; Parker *et al.*, 2005; Catani and Mesulam, 2008; Saur *et al.*, 2008; Schmahmann and Pandya, 2008; Duffau *et al.*, 2009). In contrast to the phonological and semantic factors, there were no clusters that correlated uniquely with the cognitive factor score and survived correction for multiple comparisons.

To highlight the advantages of the continuous-variable, multidimensional approach offered by PCA, we contrasted the results obtained with the extracted PCA factor scores to those found with raw test scores, conducting the SPM8 multiple-regression analysis in the same way. Of course, this necessitates selecting tests from the battery that capture the dimensions of phonology, semantics and executive-cognition. In the first ‘non-PCA selection’ analysis, we chose non-word minimal pairs, synonym judgement and Brixton spatial anticipation, as these represent widely-used ‘direct’ measures of each construct. The intercorrelations between these tests were: minimal pairs–synonyms *r* = 0.619, *P* < 0.0005; minimal pairs–Brixton *r* = 0.590, *P* < 0.0005; synonyms–Brixton *r* = 0.514, *P* = 0.003. At *P* < 0.001 voxel-level, *P* ≤ 0.001 FWE corrected cluster-level, non-word minimal pairs showed no significant clusters. Synonym judgement was uniquely associated with a significant cluster with subcortical peaks in lentiform nucleus/putamen and regions underlying left inferior frontal areas (Supplementary Fig. 1). Raven’s progressive matrices showed no significant clusters.

**Figure 6:**
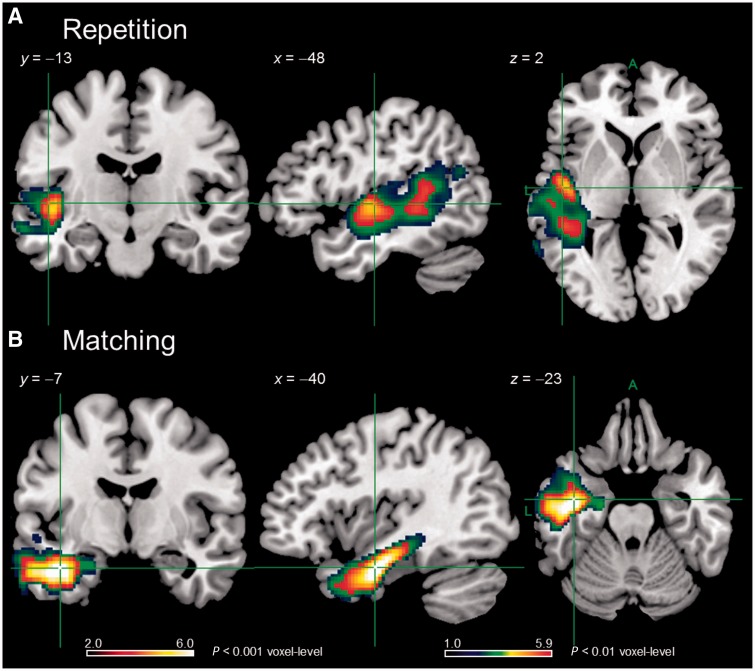
Regions found to relate significantly to delayed non-word repetition (**A**) and spoken word-to-picture matching (**B**) performance in VBCM analyses. Hot overlays are clusters significant at *P* < 0.001 voxel-level, *P* ≤ 0.001 FWE-corrected cluster-level and which were interpreted in the text. Cluster sizes 926 (**A**) and 1707 (**B**) voxels. Image threshold (*t*) 2.0–6.0. ACTC (blue/green) overlays are clusters significant at *P* < 0.01 voxel-level, *P* ≤ 0.001 FWE-corrected. Image threshold (*t*) 1.0 – 5.9.

One virtue of the rotated PCA approach is that the rotation attempts to binarize the loading of each test across the extracted factors, which helps cognitive interpretation of each factor. This also has the consequence that we can use the results of the PCA to select the individual tests that best capture the key underlying dimensions, and that are also least correlated with one another. Indeed, when we considered the individual tests with the highest loadings on each PCA factor (delayed non-word repetition, spoken word-to-picture matching, and Raven’s coloured progressive matrices) then the intercorrelations between these tests were lower, albeit still significant in some cases: repetition-matching *r* = 0.371 *P* = 0.040; repetition-matrices *r* = 0.206, *P* = 0.266; matching-matrices *r* = 0.328, *P* = 0.071. As can be seen in Fig. 6, at *P* < 0.001 voxel-level, *P* ≤ 0.001 FWE corrected cluster-level, delayed non-word repetition showed a significant cluster centred on the superior temporal gyrus, middle temporal gyrus and posterior insula, which is similar to, although more constrained than, the results seen for the phonology PCA factor. The results for spoken word-to-picture matching showed a significant cluster in the left anterior temporal lobe, although this was more extensive than that seen for the semantics PCA factor. Performance on the Raven’s was not associated with any significant clusters, similar to the results seen for the cognition PCA factor. Hence, the results using individual test scores are much stronger when PCA factor loadings have been used to guide their selection. This highlights the utility of the PCA technique to isolate tests that best capture underlying functional dimensions.

#### Lesion size

To ensure that our results were not merely attributable to lesion size, each participant’s volume was calculated from the lesion identified by the modified segmentation-normalization procedure (see ‘Materials and methods’ section). When lesion volume alone was regressed against participants’ T_1_-weighted scans a large voxel cluster in left hemisphere middle cerebral artery territory emerged as significant (Supplementary Fig. 2), representing the outer belt of the lesion overlap map (Fig. 2). The correlation between lesion volume and unrotated PCA factor score, or overall ‘severity’, was *r* = −0.545, *P* = 0.002.

For the PCA factors, lesion volume correlated relatively weakly with the phonology factor (*r* = −0.325, *P* = 0.075) and the semantic factor (*r* = −0.260, *P* = 0.159), and slightly more strongly the with executive-cognition factor (*r* = −0.411, *P* = 0.022). Crucially, including lesion volume in the VBCM model with the independent PCA factor scores did not alter the pattern of results obtained (Supplementary Fig. 3), indicating these continuous multidimensional factors are largely independent of global severity.

For the individual test analyses (*cf*. Supplementary Fig. 3 and Fig. 6), lesion volume correlated significantly with all the single tests considered in the imaging analyses: non-word minimal pairs (*r* = −0.462, *P* = 0.009), synonym judgement (*r* = −0.602, *P* < 0.0005), Brixton spatial anticipation (*r* = −0.419, *P* = 0.019), delayed non-word repetition (*r* = −0.389, *P* = 0.030), spoken word-to-picture matching (*r* = −0.410, *P* = 0.022), and identically for Raven’s progressive matrices (*r* = −0.411, *P* = 0.022). Lesion volume was included in each VBCM model with results thresholded at *P* < 0.005 voxel-level, *P* ≤ 0.01 FWE corrected cluster-level. For the non-PCA selected tests, including lesion volume removed the cluster associated with synonym judgement (i.e. no unique clusters were extracted for any of the three measures). For the tests selected on the basis of the PCA loadings, the delayed non-word repetition still showed the significant clusters for superior temporal gyrus/middle temporal gyrus and insula, spoken word-to-picture matching still showed a significant cluster centred in the left anterior temporal lobe, and Raven’s progressive matrices did not show any significant clusters (Supplementary Fig. 4). These additional test-score analyses demonstrate that more robust results emerge when individual assessments are selected according to the PCA.

#### Individual cases

**Figure 7:**
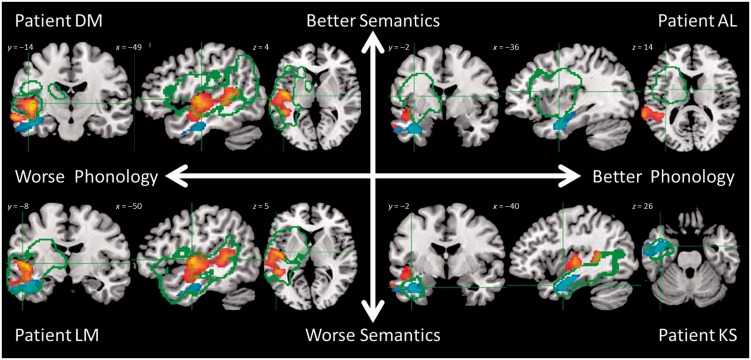
Overlap of lesion (green outline) with phonological (hot) and semantic (cool) clusters from the voxel-performance analysis for example Patients AL, DM, KS and LM. Lesion outlines were generated using Seghier *et al.*’s (2008) automated software (see main text for details). Axes reflect the participants’ scores on phonological and semantic factors of the PCA, as per Fig. 1. Patient AL = A6, Patient DM = B3, Patient LM = G2, and Patient KS = transcortical sensory aphasia in Fig. 1.

The relationship of individual patients to the group-level analyses was explored for two reasons. First, if this form of neuroscience investigation is going to have clinical utility, then it is important to explore how clearly individual behavioural and neuroimaging results relate to the maps for each language-cognitive factor. Secondly and relatedly, exemplar cases can help interpretation of the behavioural factors and their neural correlates in terms of real individual patients [given that PCA, by design, generates scores that are at least one step removed from raw clinical measures: see Lambon Ralph *et al.* (2003)]. Four exemplar participants were selected to provide contrasting pairs who scored above (‘high’) or below (‘low’) the median for the group for each language factor (Fig. 7).

In the first exemplar pair, Patient AL (high-level anomic: A6) was the patient who on average scored highest above the median on both phonology and semantics whereas Patient LM (global aphasic: G2) was the patient who on average scored in lowest below the median on both factors. This contrast provides an illustration of aphasia severity. As shown in Fig. 7, Patient AL’s lesion falls outside the key areas identified as uniquely supporting phonological and semantic processing, whereas Patient LM’s lesion largely encompasses both areas.

The second exemplar pair illustrates the specificity of impairments. Patient DM (Broca’s aphasic; B3) was the patient with the largest discrepancy between factor scores amongst those who scored ‘low’ on phonology but ‘high’ on semantics whereas Patient KS (transcortical sensory aphasia) was the patient with the largest discrepancy amongst those scoring ‘low’ on semantics but ‘high’ on phonology. As expected, Patient DM’s lesion encompassed brain regions identified as uniquely correlating with phonology, whereas those areas shown as uniquely related to semantics fell largely outside the boundary of his lesion. Conversely, Patient KS’s lesion had the opposite distribution in keeping with his transcortical sensory aphasia profile.

## Discussion

Stroke aphasia is characterized by graded impairments of multiple underlying principal language-cognitive components, with considerable variation between individual behavioural profiles (Lambon Ralph *et al.*, 2002; Schwartz *et al.*, 2006; Robson *et al.*, 2012). We adopted a novel approach to establishing the key principal language-cognitive dimensions and their neural/lesion correlates. Specifically, by completing a detailed behavioural battery across a cohort of patients with a full range of aphasia severity, we were able to apply a statistical data reduction method (PCA) to reveal the core underlying behavioural dimensions and then relate these to the distribution of the patients’ lesions. The rotated PCA revealed three language-cognitive factors: phonology, semantics and ‘executive’ cognition. Measures of aphasia severity, aphasia category and lesion size were all associated with damage to the middle cerebral artery territory as a whole. Due to the statistical independence (orthogonality) of the PCA language-cognitive components, however, we were able to identify the more specific neural regions that were uniquely associated with each core dimension of aphasia. The phonological factor explained the largest proportion of behavioural variance as one would expect from the fact that, clinically, stroke aphasia tends to be dominated by phonological impairments. Scores on this factor correlated uniquely with tissue damage in central perisylvian areas including left mid to posterior superior temporal gyrus, middle temporal gyrus, and superior temporal sulcus, Heschl’s gyrus, as well as the underlying white matter (corresponding to the arcuate fasciculus component of the dorsal language pathway). There was also a weaker relationship with damage to inferior prefrontal cortical regions (Broca’s area). In contrast, the semantic factor was uniquely related to left anterior middle temporal gyrus, and the underlying temporal stem (broadly corresponded to the ventral language route). The third factor, ‘executive’ cognition, which explained the least variance, did not uniquely covary with any brain regions in our analysis.

PCA of large behavioural data sets has been used previously in case-series studies of chronic stroke aphasic patients or comparative case-series studies (Lambon Ralph *et al.*, 2002, 2003, 2010) and other disorders such as neglect (Verdon *et al.*, 2010) but has not subsequently been related to the underlying distribution of brain damage as was done in this investigation. PCA and related approaches (where multiple tests scores are combined into single, more global measures: e.g. Schwartz *et al.*, 2009; Rapcsak *et al.*, 2009) offer at least three advantages over the use of single test measures: (i) combining multiple observations (test scores) always leads to statistically improved and more reliable measures; (ii) PCA allows decomposition of test data into the primary underlying components; and (iii) individual patient profiles can be positioned within a graded, multidimensional space (Fig. 1). Consistent with the analogy of placing colours within an RGB multidimensional space (see ‘Introduction’ section), this latter feature allowed us to retain the key differences between prototypical exemplars of different aphasia types (e.g. transcortical sensory aphasia versus conduction aphasia) but also to capture the graded variations within and between the classical aphasia types (see also Marshall, 2010), plus the many patients that clinically present with ‘mixed’ aphasia of varying severity.

The PCA-extracted dimensions also offer significant advantages over categorical classifications or individual assessment scores when investigating lesion-performance relationships. To make this explicit, we demonstrated the limitations of categorical classifications whether representing aphasia subtypes or symptom-based categories, in terms of reproducing large, overlapping neural areas that represent the same vascular territory of abnormality and thus providing minimal discrimination between the aphasia/symptom categories. Approaches that adopt these categorical classifications and then assign functional significance to the areas identified as abnormal are of limited value if only a single group of patients is considered. It should be noted that the BDAE subtypes were slightly better with respect to overlap across different groups, presumably because although these consist of exclusive categories, they are based on a multidimensional profile derived from a number of tests. In this regard, it is interesting to note that a recent large-scale VLSM analysis of stroke aphasia found very good separation between Broca’s and Wernicke’s aphasia (Henseler *et al.*, 2014). It is important to emphasize that Henseler *et al.* (2014) used predictors derived from the Aachen Aphasia Test, which provides probabilities of particular subtypes for each individual (e.g. 90% Broca’s, 10% Global), hence subtypes in this test are not exclusive or categorical but rather map to continuous variables. This work shows the value of a continuous multidimensional approach to aphasic subtypes in terms of lesion symptom mapping and here, in our use of PCA, we have shown that a similar approach can yield information concerning underlying component abilities that cut across subtypes.

To demonstrate the value of our PCA approach to lesion-symptom mapping, we contrasted these results with those obtained for individual tests. When faced with a battery of tests, one first needs to select tests that capture the underlying component abilities. In our first ‘non-PCA selection’ analysis, we chose minimal pairs non-words, synonym judgement and Brixton spatial anticipation for this purpose. As would be expected, given that no test provides a pure measure of a particular ability, these tests were highly intercorrelated and hence were not uniquely related to particular brain regions apart from a subcortical cluster for synonym judgement that was eliminated when we controlled for lesion size.

One advantage of PCA analysis is that, with rotation, there is a pressure for test loadings to be uncorrelated and associated with only one factor. Hence, one can use the factor loadings for each test to select those that best capture the underlying component abilities with maximum independence for use in VBCM analyses. In this case, these consisted of delayed non-word repetition, spoken word-to-picture matching and Raven’s progressive matrices, and they were considerably less intercorrelated than the non-PCA selected tests. Using these PCA-selected tests in VBCM not only yielded similar results to the full factor scores, but also the outcomes survived covariation of lesion volume. This analysis clearly shows the potential use of using PCA to identify the behavioural tests that most effectively capture variation along a particular underlying dimension, as identified by our interpretation of the common function reflected by each factor. Hence, while all tests necessarily draw on multiple abilities, PCA allows us to select those that provide the purest measure of a specific function. PCA can therefore be used to optimize the design of future clinical studies though highlighting the key behavioural tests of relevance and thereby minimizing the required amount of data collection.

While providing a more formal and statistically-based method for test selection in lesion symptom analyses, when used on an extensive battery of behavioural assessments, PCA also allows derivation continuous, orthogonal multidimensional measures of aphasic deficits. These scores move beyond aphasia classifications or single test scores because: (i) there is considerable severity-related shared variance across the individual assessments—thus leading to the result that much of the middle cerebral artery territory is regenerated in each lesion-performance map; (ii) individual assessments cannot be a pure measure of a single underlying language-cognitive system (e.g. naming requires semantics, phonology and motor articulation); and (iii) individual assessments will have more measurement noise than combined scores (for further discussion, see Lambon Ralph *et al.*, 2002, 2003). Because of their statistical independence, the PCA dimensions allowed us to highlight neural regions that are uniquely associated with each factor. Indeed, the unique lesion-performance maps are non-overlapping and constitute a specific subset of the entire, middle cerebral artery-dominated lesion overlap (Fig. 5). These results showed minimal change even when lesion volume was covaried out (Supplementary Fig. 3), suggesting a very good separation of the factors from overall severity. Although these results were similar to those obtained with PCA selected single tests, the factor scores revealed a wider network in the case of phonology (due inclusion of data from tests using words in addition to non-words) and a more constrained region in the case of semantics (due to inclusion of data from test with phonological output rather than input requirements). In this way, PCA yields factors that represent the optimal blend of scores across multiple behavioural tests that best capture the variance corresponding to underlying component abilities.

Before discussing each lesion-factor outcome in more detail, it is probably important to note that some parts of the middle cerebral artery-related regions (e.g. much of the lateral prefrontal cortex and inferior parietal regions) were not found in any of the unique lesion-performance maps even though they are included in the lesion overlap and aphasia-subtype/symptom group lesion maps (Figs 2 and 3) and were more likely to be damaged in patients with larger lesions (Supplementary Fig. 2). Although statistical thresholding may be a factor, the most likely explanation is that these regions are multi-functional, and support more than one of the cognitive abilities considered. Thus, for example, where regions contribute to both semantic and executive processing, such as prefrontal and some parietal regions (Thompson-Schill *et al.*, 1999; Jefferies and Lambon Ralph, 2006; Badre and Wagner, 2007; Noonan *et al.*, 2013), they will not, by definition, appear as regions that uniquely correlate with one behavioural factor, if both measures are included in the same analysis.

The phonological factor was uniquely related to tissue concentration in left posterior sylvian regions including Heschl’s gyrus, mid to posterior middle temporal gyrus, superior temporal gyrus and superior temporal sulcus, and posterior insula. These regions are consistent with those associated with phonological processing within theories framed in terms of dorsal/ventral language pathways (Hickok and Poeppel, 2004, 2007; Saur *et al.*, 2008; Ueno *et al.*, 2011; Weiller *et al.*, 2011; Kümmerer *et al.*, 2013). A number of these areas (Heschl’s, superior temporal gyrus and superior temporal sulcus) are shared between the two pathways and play a role in initial processing of auditory input, whereas more inferior regions that link this input to meaning (middle temporal gyrus) are assigned to the ventral pathway. Our patient-based results mirror the areas found to be activated during phonological processing tasks in various reviews of functional neuroimaging in neurologically-intact participants (Wise *et al.*, 2001; Hickok and Poeppel, 2004, 2007; Vigneau *et al.*, 2006; Price, 2010). In addition, repetitive transcranial magnetic stimulation to posterior superior temporal gyrus has been shown to increase error rates in language production and verbal working memory tasks (Acheson *et al.*, 2011). At a reduced statistical threshold we also found that phonological impairment in the patients was associated uniquely with damage to inferior frontal gyrus. Again, this result aligns with results from neurologically-intact participants that use functional MRI (Vigneau *et al.*, 2006; Price, 2010) or repetitive transcranial magnetic stimulation (Gough *et al.*, 2005; Hartwigsen *et al.*, 2013).

The phonological factor was also significantly related to the white matter underlying the posterior superior temporal gyrus, most likely corresponding to the arcuate fasciculus. This is consistent with the association of the arcuate fasciculus/dorsal language pathway with phonological processing (Catani and ffytche, 2005; Catani *et al.*, 2005; Parker *et al.*, 2005; Duffau *et al.*, 2008; Saur *et al.*, 2008) in studies using a variety of methodologies such as intraoperative subcortical electrical stimulation (Duffau *et al.*, 2009; Leclercq *et al.*, 2010), diffusion-weighted imaging and tractography (Parker *et al.*, 2005; Glasser and Rilling, 2008; McDonald *et al.*, 2008), VLSM (Bates *et al.*, 2003; Fridriksson *et al.*, 2010) and neuroanatomically-constrained computational models (Ueno *et al.*, 2011; Ueno and Lambon Ralph, 2013; Ueno *et al.*, 2014).

Semantic performance was found to relate uniquely to left anterior temporal lobe regions focused mainly on anterior middle temporal gyrus and the underlying temporal stem which coincides with the ventral language route and some key temporal lobe white matter tracts: the inferior longitudinal, inferior fronto-occipital, and uncinate fasciculi. Again these patient-based results mirror the findings from large-scale reviews of functional MRI studies (Vigneau *et al.*, 2006; Binder *et al.*, 2009; Visser *et al.*, 2009; Price, 2010), data from repetitive transcranial magnetic stimulation investigations (Pobric *et al.*, 2007; Woollams, 2012), direct electrical stimulation of inferior fronto-occipital fasciculus (Duffau *et al.*, 2008; Leclercq *et al.*, 2010) and neuroanatomically-constrained computational models (Ueno *et al.*, 2011). Perhaps most strikingly, this finding fits closely with the association in semantic dementia between a selective semantic impairment and anterior temporal lobe-focussed atrophy and hypometabolism (Adlam *et al.*, 2006; Butler *et al.*, 2009; Mion *et al.*, 2010), plus the outcome of a detailed analysis of semantic paraphasias in stroke aphasia (Schwartz *et al.*, 2009). Indeed, Walker *et al.* (2011) found that semantic naming errors were uniquely associated with very similar anterior temporal lobe regions once performance on executively-demanding nonverbal comprehension tasks was partialled out (which removed the involvement of additional prefrontal and parietal regions, which emerge in simple correlations between semantic error rates and tissue damage).

Various inferior parietal and inferior frontal regions have been implicated in phonological processing by functional MRI or repetitive transcranial magnetic stimulation studies (Dronkers, 1996; Vigneau *et al.*, 2006; Rauschecker and Scott, 2009; Hartwigsen *et al.*, 2010; Price, 2010), and also in controlled semantic processing by functional MRI, repetitive transcranial magnetic stimulation and targeted patient studies (Thompson-Schill *et al.*, 1999; Badre and Wagner, 2006, 2007; Jefferies and Lambon Ralph, 2006; Hoffman *et al.*, 2010, 2011; Noonan *et al.*, 2013). Overlapping areas have also been implicated in executive processing (Corbetta and Shulman, 2002; Hon *et al.*, 2006; Woolgar *et al.*, 2011; Cabeza *et al.*, 2012; Fedorenko *et al.*, 2013). Given our analyses simultaneously considered phonology, semantics and executive-cognitive functions, it is therefore not surprising that these multi-functional regions did not emerge as significant correlates of any particular ability, despite adequate lesion coverage in these areas. In terms of the absence of correlates of executive-cognitive function, it is worth noting that our test battery was not designed to extensively assess these capacities, nor did the lesions of the aphasic patients that were considered encompass many regions thought to be key for this capacity.

The general method we outline here in terms of the use of PCA to extract structure from individual behavioural profiles is of course dependent upon the data that enter the analysis. The same approach could be used over a wider battery of tests and therefore capture other factors not extensively assessed here, such as syntax and fluency. Additionally, PCA could be used to consider data from a set of tests focussing on a particular ability, such as auditory processing, and used to fractionate the component computations. Irrespective of the granularity of the PCA analysis, the current approach has the significant advantage that the underlying factors that emerge are of the ideal form for use in quantitative lesion–symptom mapping.

To conclude, the present study clearly demonstrates the utility of PCA as a means to deconstruct the multidimensionality of stroke aphasia and to establish the neural basis of the emergent language-cognition principal factors. This technique overcomes the challenges inherent in lesion-symptom mapping in patient groups with graded and variable impairments to multiple underlying functions. Our approach allows identification of discrete regions associated with individual language functions while controlling for general severity-based neural correlates. Although we have applied this method in chronic stroke aphasia, it can also be utilized in the acute phase (Kümmerer *et al.*, 2013) and in progressive cases (Henry *et al.*, 2012). This method could also be fruitfully applied to other multifaceted neurological disorders, such as Alzheimer’s disease. In addition, our use of data reduction in lesion-symptom mapping would be ideally suited to longitudinal studies charting brain changes underlying recovery in stroke or decline in dementia.

## Funding

RAB was supported by an Medical Research Council Capacity-Building PhD studentship. The research was also supported by an Medical Research Council programme grant to MALR (MR/J004146/1).

## Supplementary material

Supplementary material is available at *Brain* online.

Any requests concerning access to behavioural or imaging data should be directed to: anna.woollams@manchester.ac.uk

## Abbreviations

BDAE: Boston Diagnostic Aphasia Examination
PALPA: Psycholinguistic Assessments of Language Processing in Aphasia
PCA: principal component analysis
RGB: red-green-blue
VBCM: voxel-based correlational methodology
VBM: voxel-based morphometry
VLSM: voxel-based lesion–symptom mapping
